# Post-production protein stability: trouble beyond the cell factory

**DOI:** 10.1186/1475-2859-10-60

**Published:** 2011-08-01

**Authors:** Esther Vazquez, José Luis Corchero, Antonio Villaverde

**Affiliations:** 1 Institute for Biotechnology and Biomedicine, Universitat Autònoma de Barcelona, Bellaterra, 08193 Barcelona, Spain; 2 Department of Genetics and Microbiology, Universitat Autònoma de Barcelona, Bellaterra, 08193 Barcelona, Spain; 3 CIBER de Bioingeniería, Biomateriales y Nanomedicina (CIBER-BBN), Bellaterra, 08193 Barcelona, Spain

## Abstract

Being protein function a conformation-dependent issue, avoiding aggregation during production is a major challenge in biotechnological processes, what is often successfully addressed by convenient upstream, midstream or downstream approaches. Even when obtained in soluble forms, proteins tend to aggregate, especially if stored and manipulated at high concentrations, as is the case of protein drugs for human therapy. Post-production protein aggregation is then a major concern in the pharmaceutical industry, as protein stability, pharmacokinetics, bioavailability, immunogenicity and side effects are largely dependent on the extent of aggregates formation. Apart from acting at the formulation level, the recombinant nature of protein drugs allows intervening at upstream stages through protein engineering, to produce analogue protein versions with higher stability and enhanced therapeutic values.

## 

Aggregation and associated conformational stress of cell factories (both prokaryotic and eukaryotic) are major concerns in recombinant protein production, resulting in low yields, unstable production and limited solubility and biological activity of the products [1-9]. Basic research on protein folding and the routine implementation of several analytical procedures such as circular dichroism, mass spectrometry and infrared spectroscopy (mostly incorporated from amyloid research) [4,10-16] have expanded our understanding of how polypeptide chains cross-interact and aggregate in vivo. In bacteria, probably the most studied cell factories, aggregation as inclusion bodies, a quite common event during production of heterologous polypeptides [17,18], is now observed as a complex physiological event in which cellular agents, including chaperones [6,19,20], proteases [21-23] and actin-like proteins [24] are coordinately acting [24,25] in the frame of the cell's protein quality control machinery [26-28]. Despite aggregation as inclusion bodies might represent a source of relatively pure proteins for further refolding or extraction [29-33], or unexpectedly, a new type of nano-microparticulate biomaterials for biotechnological and biomedical applications [34-39], the use of recombinant proteins for most of biotechnological and biomedical applications requires fully soluble protein versions. A particular issue in recombinant protein aggregation is the occurrence of soluble aggregates (less apparent that large aggregates), that are being progressively recognized in production processes. These soluble clusters adopt a spectrum of forms (mainly fibrilar, spherical or amorphous) [40,41] and might be the in vivo physiological precursors and structural components of bacterial inclusion bodies [24,42]. Very different approaches have been explored at upstream, midstream and downstream levels to minimize aggregation during recombinant protein production (Figure 1). Such strategies, eventhough being mostly a trial-and-error process, often result in significant improvements of protein solubility [43-46].

**Figure 1:**
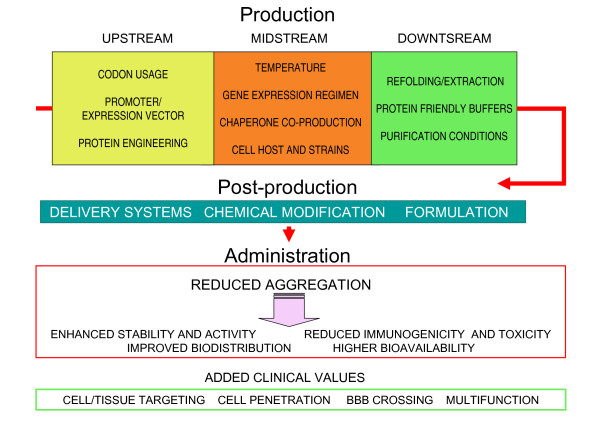
**Tool boxes through which protein solubility can be enhanced at different stages of protein production and postproduction pipelines**. Targets for improvement during *in vivo *administration are summarized in the red framed box, some of them being modulated by protein aggregation. Appropriate codon selection [82,83], using weak promoters or low copy number plasmids and protein engineering [84] are the most common upstream strategies (yellow box). Growth at sub-optimal temperatures [85,86], mild induction of gene expression, co-production of chaperones [87-89] or protein production in protease-deficient strains [90,91] or in mutants with altered redox properties [92] favor correct protein folding (orange box). Buffers and purification conditions should be optimized as per protein basis to prevent aggregation [93-97]. Alternatively, soluble protein species can be obtained by refolding inclusion body proteins [30-32] or by extracting functional proteins from inclusion bodies by mild procedures [29] (green box). Once purified, aggregation during storage or administration of protein drugs can be inhibited by appropriate excipient formulations or by chemical modification [50,60,62,98] (blue box). Also, the use of delivery systems, either through protein adsorption onto nanoparticles, nano and micro encapsulation or embedding in biocompatible materials stabilize proteins, expand their half-life in the body and permit a sustained release, resulting in enhanced bioavailability and reduced toxicity [63,64,99]. Upstream protein engineering strategies that enhance solubility during production can also affect aggregation and performance of protein drugs upon administration. Also, by this approach, novel functions that improve pharmacological performance of proteins can be gained without necessarily enhancing solubility (bottom, green framed box).

Desirably, soluble versions of recombinant proteins should keep such soluble status in post-production stages, that is, during storage and use. This need is specially acute in the case of proteins intended for therapeutic uses [47,48]. Protein drugs are commonly administered parenterally [49], what makes protein aggregation in stocks or upon administration a main concern in the Pharma industry (see for instance, http://www.eahp.eu/content/download/25193/164355/.../CoverStory20-21.pdf.pd). The high concentration at which proteins drugs are stored and administered [50] specifically favors aggregation [51]. In this context, diverse analytical procedures have been developed and specifically adapted to the detection of therapeutic protein aggregation [52-54]. Importantly, aggregation does not only render drug inactivation during storage, and fast clearance, reduction of activity, limited bioavailability and proteolytic digestion upon administration, but it also stimulates undesired immunogenicity [55]. This is a critical issue in clinics as severe side effects observed upon prolonged protein administration (as in the case of insulins, interferons, erythropoietin and growth hormone) are antibody-dependent [56-59].

Chemical modification of proteins and the use of appropriate excipients (Figure 1) are the most taken approaches for protein drug stabilization [60-62]. Obviously, emerging concepts in Nanotechnology, Nanomedicine and in Material Sciences offer new biocompatible vehicles for protein encapsulation or embedding, mainly at the nanoscale, through which the stability, tissue targeting and bioavailability during drug delivery are dramatically enhanced [63,64]. Many among those such nanostructured materials are from bacterial origin [65].

Being proteins flexible molecules suitable to be re-designed by genetic methods, upstream protein engineering, one of the main approaches to prevent aggregation during production (Table 1, up), is also useful to stabilize protein drugs during use (Table 1, bottom). In addition, modification of the protein primary sequence permits a fine tuning of protein features such as oligomerization, activity, cell targeting and cell penetration, that represent additional values in the performance of a protein drug (Table 1; Figure 1, bottom). In this regard, protein engineering is revealed as an extremely flexible approach to enhance the stability of proteins during production, storage and use, but also to improve their performance in *in vivo *uses. Reduction of aggregation is expected to minimize immunogenicity, increase proteolytic stability, improve bioavailability and limit side-effects, as aggregation has a pivotal role in all these issues [55,59,66-69]. In addition, protein modification can offer added values to protein drugs, by conferring novel functions that improve pharmacological performance without necessarily enhancing solubility (Figure 1, green framed box). These include cell or tissue targeting or enhanced cell penetration by the fusion to a cell receptor ligand or an antibody [70-74], enhancing half-life and bioavailability by fusion to transferrin [75], albumin [76], or albumin-binding peptides [77] and crossing the brain-blood barrier (BBB) by the incorporation of cationic peptides [78]. Creating multifunctional proteins by the appropriate combination of protein domains in a single polypeptide chain is being especially explored for the construction of protein-only artificial viruses, in which the therapeutic nucleic acids are encapsulated by chimerical protein building blocks [72,79-81]. Further exploration of protein engineering focused on post-production issues is strongly required and it should allow the emergence of optimized drugs to fulfill their increasing demand.

**Table 1:** Protein engineering strategies to reduce aggregation or derived effects during either production or administration, illustrated by representative examples.

Protein engineering strategy	Result	Protein	Reference
***Improving protein folding during production***			

Cys→Ser point mutations	Reduced aggregation, enhanced proteolytic stability	bFGF^a^	[100]
Point mutations in an hydrophobic stretch	Reduced aggregation	11 beta-HSD1	[101]
Directed evolution/point mutations	Reduced aggregation	Cytochrome P450sca-2	[102]
Fusion of SUMO tag	Improved refolding	Fgf15	[103]
Polycationic amino acid tag fusion	Reduced aggregation	*Candida antarctica *lipase B	[104]
Fusion to polylysines or polyarginines	Reduced aggregation	BPTI-22	[105]
Fusion to MBP	Reduced aggregation	Ribonuclease inhibitor	[106]
Fusion to GrpE	Reduced aggregation	hIL-3	[107]
Fusion to NusA	Reduced aggregation, enhanced proteolytic stability	E8R viral protein	[108]
***Improving protein folding, stability and performance during administration***			

Single amino acid substitution	Inhibited oligomer formation; enhanced bioavailability	Insulin Aspart ^®^	[109]
Single amino acid substitution	Improved folding	INF-β-1b (Betaferon ^®^)	[110]
N-terminal peptide deletion	Enhanced stability	KGF	[111]
Fusion with albumin	Extended half-life	Albinterferon α-2b	[76]
Fusion with transferrin	Enhanced gastrointestinal adsorption	hGH	[75]
Artificial consensus protein sequence	Enhanced activity	Interferon αcon-1 (Infergen ^®^)	[112]
Fusion of a HIV Tat segment	Enhanced solubility	p53	[113]
Fusion of a HIV Tat protein and ODD	Enhanced stability and activity in hypoxic tumor cells	Casp-3	[114]
Fusion of a HIV Tat protein and sequence modification	Cell penetration and selective activation in HIV-infected cells	Casp-3	[115]
Ligand incorporation (mainly antibody fragments)	Enhanced stability and bioavailbility	IL-2	[71]

^a ^Abbreviations are: 11 beta-HSD1, 11 beta-Hydroxysteroid dehydrogenase type 1; aFGF, acidic fibroblast growth factor; bFGF: Fgf15, Fibroblast growth factor 15; BPTI-22, Bovine pancreatic trypsin inhibitor variant 22; Casp-3, caspase 3; HIV, human immunodeficiency virus; hFGF, Human basic fibroblast growth factor; BSA, bovine serum albumin; HAS, human serum albumin; hGH, human growth hormone; hIL-3, human interleukin-3; KGF, keratinocyte growth factor; IL, interleukin; MAGOH, Protein mago nashi homolog; MBP, maltose-binding protein; OOD, oxygen-dependent degradation domain of hypoxia-inducible factor-1alpha; rhDNase, recombinant human DNAse; SUMO, small ubiquitin-related modifier.

## Conclusions

Stability and solubility of recombinant proteins is a critical issue at both production and post-production stages. For a biomedical use of proteins as pharmaceuticals, high solubility not only supports stability but it also enhances bioavailability and reduces immunogenicity and undesired toxic effects. Among other approaches to stabilize protein drugs, such as chemical modification, proper formulation and encapsulation, protein engineering is a very flexible route to improve protein folding during production and reduce aggregation during storage and *in vivo*. Furthermore, the modification of protein primary sequence permits to confer additional functional values, such as binding to serum albumin, binding to cell surface receptors and cell membrane (or BBB) crossing, thus improving biodistribution, expanding the half-life and enhancing the biological potential of the drug. The wide spectrum of possibilities of post-production-addressed protein engineering is probably to be yet realized.

## Competing interests

The authors declare that they have no competing interests.
